# The Effects of Past COVID-19 and Vaccination on Antibody Levels, Cellular Immunity, and Cytokine Production by Peripheral Blood Mononuclear Cells

**DOI:** 10.3390/biomedicines14040923

**Published:** 2026-04-17

**Authors:** Yulia A. Desheva, Tatiana V. Gupalova, Polina A. Kudar, Galina F. Leontieva, Igor V. Kudryavtsev, Andrey S. Trulioff, Danila S. Guzenkov, Victoria A. Matyushenko, Elena A. Bormotova, Daniil D. Sokolovsky, Georgy A. Matveev, Boris P. Nikolaev, Alexander N. Suvorov

**Affiliations:** 1 Federal State Budgetary Scientific Institution “Institute of Experimental Medicine”, Academician Pavlova St., 12D, St. Petersburg 197022, Russia; tvgupalova@rambler.ru (T.V.G.); galeonte@yandex.ru (G.F.L.); bormotovae@rambler.ru (E.A.B.);; 2 Technopark of St. Petersburg, Prospekt Medikov, 3, lit. A, St. Petersburg 197022, Russia; 3 Institute of Cytology of the Russian Academy of Sciences (RAS), St. Petersburg 194064, Russia

**Keywords:** SARS-CoV-2, spike protein, nucleocapsid protein, recombinant viral antigens, long-term immune response, antigen-specific cellular immunity, immune memory, cytokine profile, PBMC stimulation, IFN-γ, IL-12p70, IL-10, TNF-alpha

## Abstract

**Background/Objective:** This study is a cross-sectional investigation of long-term immune responses measured at different time intervals after COVID-19 infections, vaccinations, or combined exposure. The focus is on immune reactivity against recombinant spike (S) and nucleocapsid (N) protein antigens. **Materials and Methods:** Serum antibody levels were assessed up to four to four and a half years after infection or immunization, including virus-specific immunoglobulin G (IgG), IgA and IgM antibodies, as well as neutralizing antibodies against the S-protein. Cellular immunity was assessed by analyzing peripheral blood mononuclear cells (PBMC; *n* = 43 in first cohort, *n* = 32 in second cohort), including T-helper memory and cytotoxic subsets, and cytokine production after in vitro stimulation with recombinant SARS-CoV-2 proteins. A multiplex cytokine assay was used to analyze effector and regulatory immune responses. **Results:** Virus-specific IgG antibodies persisted for years after exposure to SARS-CoV-2, with IgG against the receptor-binding domain (RBD) correlating most strongly with neutralizing activity. Vaccinated individuals demonstrated higher IgA responses, whereas antibodies to the N-protein were associated with previous infection. No IgM antibodies were detected in any subjects, suggesting an immune response based on memory rather than ongoing infection. PBMCs from individuals with a history of both COVID-19 exposure and vaccination exhibited enhanced responsiveness, characterized by increased frequencies of memory T cells compared to vaccination alone. Stimulating with the S-protein induces higher cytokine production, including IFN-gamma, TNF-alfa, and IL-12(p70), compared with stimulation by the N-protein. Cytokines such as IL-10 and TGF-beta are also elevated, suggesting immune regulation rather than persistent inflammation. **Conclusions:** SARS-CoV-2 infection and vaccination are associated with persistent humoral and cellular immune responses detectable several years after exposure. Individuals with hybrid immunity exhibit broader and functionally enhanced immune reactivity, indicating more robust long-term immune memory. Future studies should focus on the long-term consequences of hybrid immunity and optimize other vaccine strategies, including recombinant antigen vaccines.

## 1. Introduction

Recombinant viral antigens are widely used in modern vaccines and diagnostic platforms, including those developed for severe acute respiratory syndrome coronavirus 2 (SARS-CoV-2) [1,2]. Although the short-term and mid-term immunogenicity of these vaccines has been studied extensively, much less is known about their long-term effects on the quality of antigen-specific immune memory induced by natural infections, vaccinations, or a combination of both [3,4]. In particular, there is insufficient information about the persistence and functional properties of humoral and cell-mediated immune responses to specific viral proteins several years after exposure to the antigen [5].

Among SARS-CoV-2′s structural proteins, the spike (S) and nucleocapsid (N) proteins represent immunologically distinct antigens [6,7]. The S-protein mediates viral entry via interaction with the ACE2 receptor and is the main target of neutralizing antibodies, as well as being the only antigen encoded by most licensed vaccines [8]. In contrast, N is highly conserved and abundantly expressed, eliciting robust T-cell responses but not inducing virus-neutralizing antibodies [9,10]. As a result, responses to S and N provide complementary information on vaccine-induced immunity, allowing discrimination between different exposure histories.

Previous studies have demonstrated that SARS-CoV-2–specific IgG antibodies and antigen-responsive T cells can persist for at least 12 months following acute infection [11,12,13]. However, data beyond this time frame remain limited, and direct comparisons of long-term immune responses to S-and N-proteins at both humoral and cellular levels are scarce. Moreover, the majority of available studies focus on measuring antibody titers rather than functional cellular immunity, such as memory T-cell subsets and cytokine responses following antigen stimulation. Long-term studies are therefore essential to better understand sustained immune alterations, including potential immune exhaustion or autoimmune processes, and to inform revaccination strategies. To address these gaps, the present study aimed to characterize long-term humoral and cellular immune responses to recombinant SARS-CoV-2 S-protein and N-protein in individuals with different exposure histories, including prior infection, vaccination, or hybrid immunity. We analyzed antibody profiles, virus-neutralizing activity, and antigen-specific cellular responses of peripheral blood mononuclear cells (PBMCs) following stimulation with recombinant viral antigens. By examining participants several months to several years after infection and/or vaccination, this cross-sectional analysis provides insight into the durability and functional characteristics of SARS-CoV-2–specific immune memory. In addition, a bioinformatic analysis of recombinant S- and N-proteins was performed to predict potential T-cell epitopes and to support the selection and interpretation of antigens used in functional cellular assays.

## 2. Materials and Methods

### 2.1. Ethics Statement

The study was approved by the Ethics Committee of the Institute of Experimental Medicine (protocol 1/23 dated 20 April 2023). All patients signed informed consent forms.

### 2.2. Bioinformatics Analysis

Amino acid sequence analysis was performed using Ugene [14]. MHC class I binding predictions were performed using the NetMHC 4.0 web server (DTU Health Tech, Lyngby Denmark; https://services.healthtech.dtu.dk/services/NetMHC-4.0/ accessed on 29 November 2025) [15,16]. Analysis was restricted to 9-mer peptides against a reference set of 12 HLA supertype representatives (HLA-A01:01, A02:01, A03:01, A24:02, A26:01, B07:02, B08:01, B15:01, B27:05, B39:01, B40:01, B58:01). Only peptides classified as strong binders (SB) were selected, using the default threshold of %Rank < 0.5. Structure prediction was performed using AlphaFold 3 [17]. The bioinformatic analysis was performed as a supportive characterization of the recombinant antigens, providing information on predicted epitope content and potential immunogenicity, rather than as a direct analysis of patient-specific immune responses.

### 2.3. Patients

The study represents a cross-sectional analysis of immune parameters measured at a single sampling time point, with participants differing in the time elapsed since SARS-CoV-2 infection and/or vaccination.

The study included two cohorts of patients. Participant groups and study design are indicated in Table 1.

The first cohort covered the period from one year up to 4.5 years after COVID-19 infection on average. As shown in Table 1, the study participants were divided into three groups based on their medical histories:

1. Group 1—individuals who were not vaccinated;
2. Group 2—vaccinated individuals who had not had acute COVID-19
3. Group 3—vaccinated and had had acute COVID-19.

Participants in groups 2 and 3 experienced COVID-19 with unknown severity, and their medical records included PCR tests and documented histories prior to clinical diagnosis of COVID-19. Group 3 included vaccinated individuals who, based on their medical history, had COVID-19. The medical histories showed that there were no symptoms of post-COVID syndrome in patients who had recovered from acute COVID-19 (groups 2 and 3).

The second cohort spanned the period between 0.5 and 3 years post-COVID-19 infection.

Due to the retrospective study design and limitations in confirming repeated infections, participants were not stratified according to the number of COVID-19 episodes, but were classified based on the presence or absence of a prior infection history.

Handling of time since infection/vaccination. Time since SARS-CoV-2 infection or vaccination was treated as a retrospective categorical and continuous variable based on documented medical history. All blood samples were collected during a single sampling period (November 2024). Therefore, immune parameters reflect cross-sectional measurements obtained at different intervals after exposure rather than longitudinal follow-up of the same individuals.

### 2.4. Vaccine

The patients were vaccinated using the GAM-COVID-Vac vaccine (also known as Sputnik V), an adenoviral vector vaccine that expresses the full-length glycosylated S-protein of SARS-CoV-2. Its effectiveness, determined by the induction of specific antibodies on the 42nd day after vaccination, is 91% [18]. Because the study was retrospective and vaccination histories were obtained from medical documentation and participant reports, detailed information about booster doses and exact vaccination schedules was not available for all participants.

### 2.5. Obtaining Blood Serum Samples

Serum samples were obtained from venous blood collected from patients in November 2024 using a standard procedure. After clotting, the samples were centrifuged for 20 min at 1000× *g*, and then the supernatants were frozen and stored at −70 °C.

### 2.6. Determination of the Level of Antibodies to SARS-CoV-2

The level of antibodies (IgG, IgA, IgM) to the SARS-CoV-2 virus in blood serum samples was determined by ELISA using the SARS-CoV-2-IgG-ELISA-BEST kit manufactured by Vector-BEST, Novosibirsk, Russia. Commercial enzyme-linked immunosorbent assay kits for the detection of IgG/M/A antibodies to SARS-CoV-2 from AO “Vector-Best” (Novosibirsk, Russia) were used. These kits are designed for the qualitative detection of IgG/M/A antibodies to SARS-CoV-2 in human serum or plasma using solid-phase ELISA according to the manufacturer’s instructions. To calculate the positivity coefficient (PC), the critical value of optical density (OD) was calculated using the formula: ODcrit = ODav K (-) + 0.2, where ODavK (-) is the average optical density of the negative control samples. The antibody level in the sample was expressed as an optical density (OD) using the formula: OD = ODbr/ODcrit, where ODbr is the optical density of the analyzed sample. The test result was considered positive if OD ≥ 1.1, negative if OD < 0.8, and borderline if 0.8 ≤ OD < 1.1.

### 2.7. Concurrent Microneutralization Assay

The SARS-CoV-2 surrogate virus neutralization assay (AtaGenix, Wuhan, China), a variant of a competitive ELISA, detects circulating antibodies that neutralize the interaction between SARS-CoV-2 and its receptor ACE2. The assay uses a protein-protein binding to detect antibodies that bind to the receptor-binding region (RBD). The neutralization percentage is calculated based on the OD450 values according to the manufacturer’s instructions.

### 2.8. Microneutralization (MN) Assay in Cell Culture

The protocol of microneutralization was approved and published previously [19]. Vero CCL-81 cells were seeded at a density of 4 × 10^4^ cells per well in 96-well plates with 150 μL of DMEM containing 10% fetal bovine serum (FBS, Corning, Kaiserslautern-Wiesbaden, Germany). The cells were incubated overnight at 37 °C and 5% CO_2_ to allow them to form a monolayer with 95–100% confluence. Serum samples (64 μL) were preheated at 56 °C for 30 min and stored at −20 °C if necessary. Serial two-fold dilutions of serum samples were carried out using a conditioned medium containing 2% FBS, starting from an initial dilution of 1:10. Control rows contained only conditioned medium. The SARS-CoV-2 isolate hCoV-19/St_Petersburg-3524S/2020 (GISAID EPI_ISL_415710) belonging to the Wuhan lineage was used in the study. The work stock of the virus was titrated by the standard method to determine a 50% infection dose in tissue culture by sequential 10-fold dilutions of the virus, which reached a titer of 10^7^ TCID_50_/mL. Cytopathic effect (CPE) was then detected by ELISA using an N-specific monoclonal antibody [20]. 55 µL of prepared serum serial dilutions were transferred to round-bottom immunological reaction wells, then 55 uL of virus with titer of 300 TCID/5 μL was added. The plates were incubated 1 h at 37 °C. The maintenance medium was removed from the cell culture plates, then 100 µL virus/serum mixtures were added to cells and incubated again for 1 h at 37 °C and 5% CO_2_. The contents of the cell culture plate with the virus and serum mixture were completely removed, after which 100 µL of serum dilutions were added to the corresponding wells of the culture plates and incubated for another 48 h at 37 °C and 5% CO_2_. After 48 h, cells were examined for monolayer integrity and CPE using microscopy. Wells with non-specific cell lysis were marked separately and removed from analysis.

The medium was removed, and 100 µL of 10% formalin in PBS was added. Plates were incubated at 4 °C for 24 h, then washed with PBS and transferred to a BSL-2 laboratory for further analysis. Then, plates were washed with PBS, permeabilized with 100 µL of 1:1000 Triton X-100 in PBS for 15 min at room temperature. After washing, 100 µL of 3% skim milk in PBS was added for 1 h at 37 °C. Primary mouse antibodies against the N-protein of SARS-CoV-2 (50 µL, 2.0 µg/mL) were added for 1 h at 37 °C, followed by washing and addition of secondary antibodies against rabbit IgG conjugated with horseradish peroxidase (50 µL, 1:2000) for 1 h at 37 °C. Plates were washed again, dried, and incubated with TMB-Ultra substrate (50 µL, Thermo Fisher, Waltham, MA, USA) for up to 20 min in dark. The reaction was quenched with 25 μL 1M sulfuric acid.

Results were analyzed using a BIORAD xMark Microplate spectrophotometer (Bio-Rad Laboratories, Inc., Hercules, CA, USA), Microplate Manager^®^ 6 Software at 450 nm. The 50% inhibitory activity values were calculated using four-parameter nonlinear regression analysis.

### 2.9. Recombinant Proteins of SARS-CoV-2

Recombinant proteins of the SARS-CoV-2 virus were obtained using *Escherichia coli* strains DH5a and BL21 as recipients for transformation as described previously [21,22]. The recombinant fragment corresponds to a conserved central region of the N-protein and differs from the Wuhan-Hu-1 reference sequence only by a single Asn insertion outside the SR-rich immunodominant region. Therefore, there is no significant change in antigenic determinants [21]. The S-protein sequence corresponded to the XBB.1.5” (Kraken) variant and includes part of the RBD domain [22].

### 2.10. Enzyme-Linked Immunosorbent Assay (ELISA) for the Determination of IgG Subclasses in Blood Serum Using Recombinant Peptides

Determination of antibody subclasses to recombinant peptides of SARS-CoV-2 was performed as previously described [23]. For the experiment, Nunc MaxiSorp 96-well plates (Thermo Fisher Scientific, Waltham, MA, USA) were coated with 2 μg/mL of recombinant proteins. Before antibody detection, serum samples were heated to 56 °C for 30 min. Following three rounds of washing, serial dilutions of the sera were added to the coated wells in 1:4 increments. After a 1.5 h incubation at 37 °C, the wells were washed again to remove unbound antibodies. To detect the bound antibodies, horseradish peroxidase-conjugated rabbit anti-human IgG antibodies (Polygnost, Leningrad Region, Russia) were used. The final ELISA titers were determined by identifying the highest dilution where the optical density at 450 nm (OD450) surpassed the mean OD450 plus 3 standard deviations (SD) of the negative control wells. The mean values for the control wells were calculated for each dilution using 4 to 6 pre-SARS-CoV-2 negative blood sera.

### 2.11. Obtaining and Incubating PBMCs

Blood collection and isolation of peripheral blood mononuclear cells (PBMCs) were performed during November 2024 using a standard method [24]. Heparinized (10 U /mL) whole blood was diluted 1:2 with phosphate-buffered saline (PBS) without calcium and magnesium. Aliquots of approximately 20 mL of the diluted cell preparations were transferred to sterile 50 mL conical tubes. Then, 12 mL of Histopac-1077 was applied to the diluted cell preparation and the tubes were centrifuged without braking at 2400 rpm (~800× *g*) for 20 min at room temperature. PBMCs were removed using a sterile pipette and transferred to a sterile 50 mL conical tube. The cells were washed twice to a volume of 50 mL with PBS and centrifuged at 1700 rpm (~500× *g*) for 5 min. The cell pellet was resuspended in RPMI 1640 medium containing 10% fetal calf serum with antibiotics.

### 2.12. Stimulation of PBMC with Recombinant SARS-CoV-2 Virus Proteins

A fraction of mononuclear cells was isolated from peripheral blood and diluted in complete RPMI-1640 culture medium (BioloT LLC, St. Petersburg, Russia) supplemented with 10% inactivated fetal bovine serum (FBS, Gibco Life Technologies, Carlsbad, CA, USA), 50 μg/mL gentamicin (BioloT LLC, Russia), and 2 mM L-glutamine (BioloT LLC, St. Petersburg, Russia). 100 μL of the cell suspension (at a concentration of 1–2 × 10 ^7^ cells/mL) was added to the wells of a 96-well plate. Purified protein derivative (PPD) of mycobacteria at a final concentration of 5 μg/mL served as a biological control. To stimulate the cells, the antigens under study (N- and S-proteins from SARS-CoV-2 virus) were used at a final concentration of 5 μg/mL. In addition, sterile PBS was added to some wells as controls since sterile PBS was used for PPD and N- and S-proteins dilution. The samples were then incubated at 37 °C in an atmosphere of 5% CO_2_ for 18 h.

### 2.13. The T Cell Antigen-Specific Immune Response

The T cell antigen-specific immune response was assessed using an ICS (intracellular cytokine staining) assay. After incubation for 18 h at 37 °C and 5% CO_2_, 50 µL of RPMI-1640 medium with Brefeldin A (cat.No 420601, Biolegend, San Diego, CA, USA), diluted 1:250 was added to each well, followed by incubation for another 5 h at the same conditions. The cells were then centrifuged at 500× *g* for 5 min at 4 °C for 5 min, and stained with a surface antibody cocktail (PerCP/Cyanine 5.5 anti-human CD45RA (cat. 304122, clone HI100), PE/Cyanine7 anti-human CD197 (CCR7) (cat. 353204, clone G043H7), APC anti-human CD4 Antibody (cat. 300514, clone RPA-T4), Alexa Fluor^®^ 700 anti-human CD8 Antibody (cat. 344724, clone SK1), APC/Fire™ 750 anti-human CD3 Antibody (cat. 344840, clone SK7), and Zombie Aqua™ Fixable Viability Kit (cat. 423102)) at 4 °C for 20 min in the dark for detection live/dead cells. All antibodies were manufactured by Biolegend (San Diego, CA, USA). Fixation and permeabilization were conducted using Cyto-Fast™ Fix/Perm Buffer Set (Biolegend, San Diego, CA, USA), according to the manufacturer’s recommendations, followed by staining of PBMC samples with FITC anti-human IFN-γ (cat. 502506, clone 4S.B3), PE anti-human IL-2 (cat. 310610, cat. W19046A), and Brilliant Violet 421™ anti-human TNF-α (cat. 502932, clone MAb11) antibodies for another 20 min. Finally, after two wash steps (500 g, 4 °C for 5 min) the cells were fixed in 100 µL of Cyto-Last™ Buffer (Biolegend, San Diego, CA, USA) and stored in a dark cool place prior to the flow cytometric analysis. Data acquisition was performed with a Navios flow cytometer (Beckman Coulter, Brea, CA, USA), and at least 500,000 events were measured per each sample. The results were analyzed using Kaluza Analysis 2.1 (Beckman Coulter, Brea, CA, USA). The proportion of antigen-specific T cells was calculated by subtracting the negative control from the cytokine-positive CD4+ and CD8+ T cells. The gating strategies for antigen-specific CD4+ and CD8+ T cells are shown in Supplementary Figures S1 and S2.

### 2.14. Determination of Cytokine Concentrations

Cytokine concentrations (pg /mL) were determined using a multiplex flow cytometer assay with the LEGENDplex ™ HU (13-Plex Panel) kit from Biolegend (USA), which allows for the determination of concentrations of 13 human cytokines (IL-4, IL-2, CXCL10 (IP-10), IL-1β, TNF, CCL2 (MCP-1), IL-17A, IL-6, IL-10, IFN-γ, IL-12p70, CXCL8 (IL-8), and free active TGF-β1) according to the manufacturer’s protocol. The high sensitivity of this method allows for the determination of the cytokines in a wide concentration range. The LEGENDplex^TM^ data software, version 8 package (BioLegend, San Diego, CA, USA) was used to analyze the results. When stimulated with SARS-CoV-2 coronavirus proteins, individuals who responded to stimulation were defined as samples in which antigen-induced cytokine-positive T-cell frequencies exceeded the corresponding unstimulated control above a predefined threshold, accounting for background variability.

### 2.15. Statistical Analysis

Statistical data processing was carried out using the Prism 8 software package (GraphPad software, v10.4.0, San Diego, CA, USA). Medians (Me) and lower and upper quartiles (Q1; Q3) were calculated and used to present the antibody response and blood test levels. The geometric mean titers (GMT) were used to express the average values of antibody titers. The normality of distributions was studied using D’Agostino & Pearson omnibus normality test. When comparing samples that did not meet the assumptions of normal distribution of the dependent variable within each group and homogeneity of variance, a nonparametric test was used: for multiple comparisons, Friedman’s test (F-test (ANOVA) or Kruskal–Wallis (Kruskal–Wallis ANOVA). To assess intergroup differences, we used the Mann–Whitney (Mann–Whitney U test) or the Wilcoxon matched-pairs signed rank test. For nominal data, we used a two-tailed version of Fisher’s exact test. A nonparametric measure of the statistical relationship between two variables was performed using Spearman’s rank correlation coefficient (r). A *p*-value of <0.05 is considered statistically significant.

## 3. Results

### 3.1. Results of Bioinformatics Analysis

The purpose of this analysis was to demonstrate the theoretical capacity of the recombinant proteins to contain T-cell epitopes with broad HLA coverage. The results are presented in Table 2.

All identified epitopes are peptides presented by major histocompatibility complex class I (MHC-I) molecules and may be presented to CD8+ T cells (Table 3 and Table 4).

This means that these epitopes are intended for recognition by CD8^+^ cytotoxic T lymphocytes, which destroy infected cells [25]. The recombinant N-protein is characterized by a narrow allelic cover, as its epitopes are limited to only two alleles (HLA-B*07:02 and HLA-B*39:01), which reduces the effectiveness of the population—protection will be strong only for carriers of these two alleles [15]. The S-protein, on the other hand, is characterized by broader allelic coverage with epitopes expressed through multiple common alleles of HLA-A and HLA-B, providing protection for most of the population.

### 3.2. Humoral Immunity

**Figure 1:**
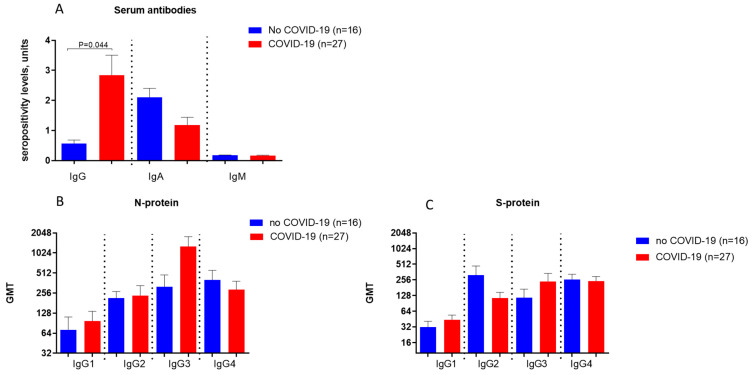
Antibodies to SARS-CoV-2 were examined in different patient groups after 2–4.5 years after COVID-19. The dashed lines in the diagrams separate antibody classes/subclasses. (**A**). Antibody classes against SARS-CoV-2 were determined using commercial assays. (**B**). IgG subclasses to the recombinant N-protein. (**C**). IgG subclasses to the recombinant S-protein.

Determination of the levels of different classes of antibodies (IgG, IgA, and IgM) against the SARS-CoV-2 virus in blood serum samples among the first cohort of patients was performed using commercially available test systems as described in the Materials and Methods. Patients were examined 2–4.5 months after the onset of COVID-19 symptoms. Seropositivity levels were expressed in arbitrary units as specified by the manufacturers. Patients without a history of COVID-19 exhibited significantly lower virus-specific IgG levels compared to individuals who had experienced COVID-19 (Figure 1A).

IgG levels among participants in the group of those patients who did not have COVID-19 are low, below the seropositivity threshold (1.2). This may confirm the lack of previous contact with SARS-CoV-2 (Figure 1A). Differences in serum IgG levels between patients who had not had COVID-19 and patients after COVID-19 were statistically significant (*p* = 0.0135). The presence of IgA antibodies in individuals presumed not to have been exposed to SARS-CoV-2 may be explained by the high proportion of vaccinated individuals (84.5%).

We used the recombinant N and S-proteins of the coronavirus to identify antibody subclasses to the SARS-CoV-2 virus (Figure 1B,C). Elevated mean IgG levels for the N-protein were found among individuals who had COVID-19, with IgG3 levels being particularly elevated (Figure 1B). The highest IgG2 levels to the S-protein were found in those who had no history of COVID-19. This may be explained by the fact that coronavirus vaccines target the S-protein of the SARS-CoV-2 coronavirus, which was present in the majority of those who did not have COVID-19 (Figure 1B). IgG4 to the N and S-proteins did not differ significantly between individuals who had COVID-19 and those who had not (Figure 1B,C).

**Figure 2:**
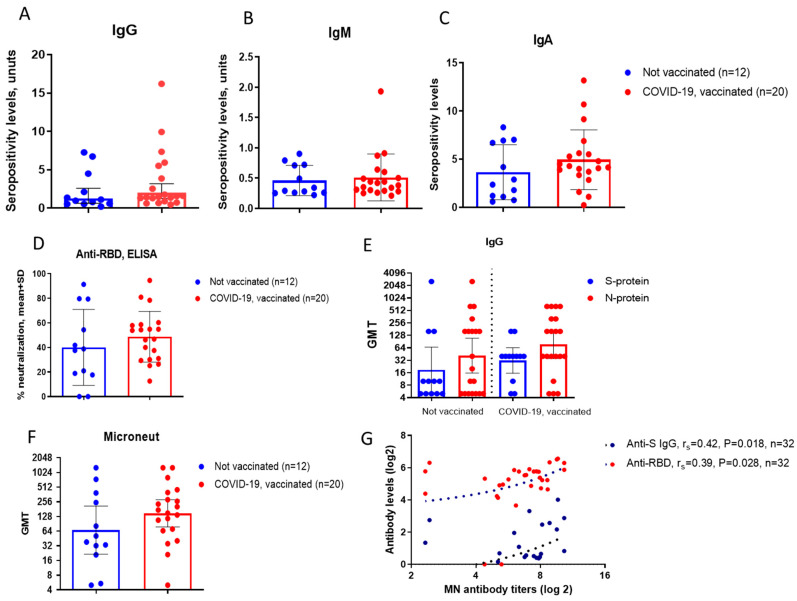
Antibodies to SARS-CoV-2 in patients examined after 0.5–2.5 years post COVID-19 (2nd cohort). (**A**–**C**). The mean seropositivity level of IgG, IgA and IgM was determined using commercial kits. (**D**). Anti-RBD antibodies were detected using concurrent ELISA. (**E**). ELISA was used to detect IgG antibodies to the recombinant N-protein and S-protein of SARS-CoV-2. (**F**). Neutralizing antibody titers were determined in a microneutralization assay using SARS-CoV-2 virus in cell culture. (**G**). The correlation between neutralizing antibody titers and anti-SARS-CoV-2 IgG or anti-RBD was analyzed using Spearman’s correlation coefficient (r_s_).

The second cohort consisted of patients with a post-disease period ranging from six months to three 2.5 years. In this cohort, only four individuals had no history of COVID-19. We compared antibody levels in unvaccinated persons and in vaccinated individuals. Mean levels of IgG, IgA and anti-RBD antibodies were higher among vaccinated persons compared to non-vaccinated (Figure 2A–D), as well as other types of antibodies, such as IgG to N- and S-protein or neutralizing antibodies (Figure 2E,F).

A statistically significant relationship of moderate strength was observed between neutralizing antibodies with anti-SARS-CoV-2 IgG and anti-RBD (Figure 2G).

### 3.3. Cellular Immunity

PBMCs from 43 patients in 1st cohort were stimulated with N- and S-proteins for 24 h, and immune cell response and cytokine production were estimated. The number of patients from the 1st cohort who responded to PBMCs stimulation with the recombinant N- and S-proteins are presented in Table 5 and Table 6.

In regard to cytotoxic CD8+ memory T cells, this cell population is strongly activated in patients who had recovered from COVID-19 and had subsequently been vaccinated (Table 6).

**Figure 3:**
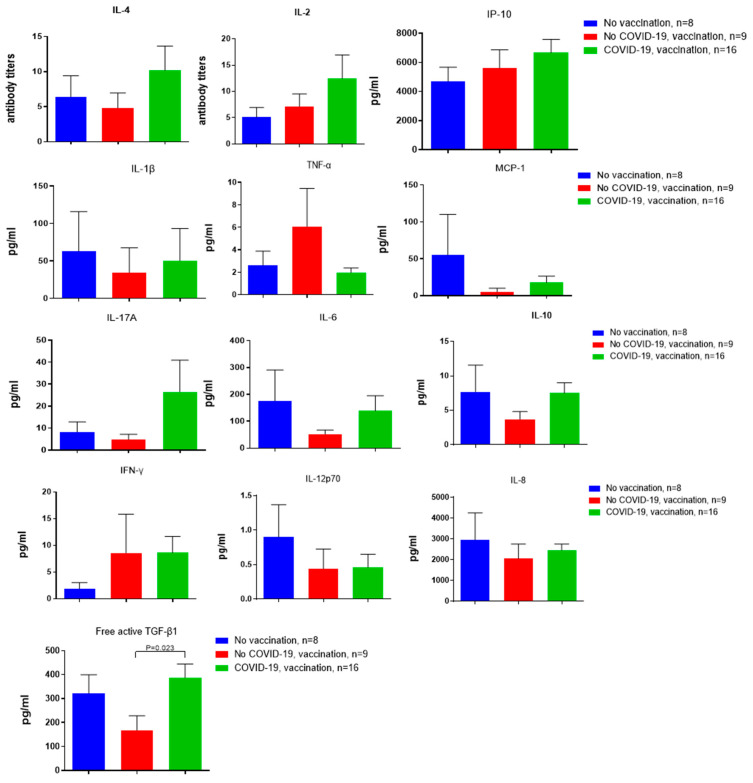
Spontaneous cytokine production by PBMCs from different patient groups, 1st cohort. (*n* = 33). *p* values were calculated using the Mann–Whitney test.

Figure 3 presents the results of a study of spontaneous cytokine production by peripheral blood mononuclear cells among three groups of patients from the 1st cohort. For technical reasons, 10 samples from the previously analyzed cohort of patients were excluded from the analysis.

### 3.4. Cytokine Responses

Previous COVID-19 in combination with vaccination was associated with sustained production of cytokines related to both immune activation and immune regulation. These include IL-2 and IL-17A, and regulatory cytokines such as IL-10 and TGF-β, by peripheral blood mononuclear cells, as well as a decrease in production of TNF-α, IL-1 β, IL-6, MCP-1 (Figure 3).

**Figure 4:**
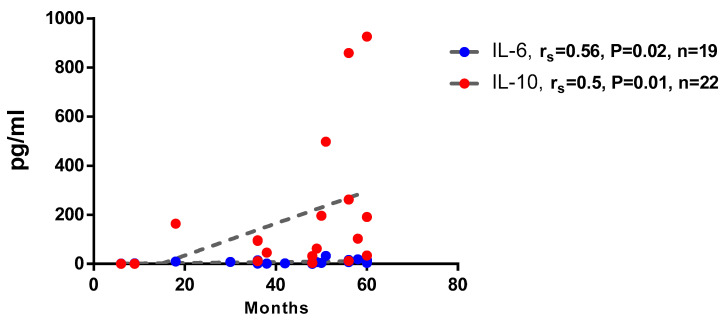
Correlation analysis of the spontaneous production of cytokines by the PBMCs, depending on the time after the disease (in months). The points in the plot represent individual participants, with coordinates that correspond to the measured values for the indicated parameters: the number of months since the disease, and the levels of IL-6 and IL-10, respectively.

Interestingly, there was a statistically significant moderate positive association between the months since COVID-19 and the spontaneous production of the proinflammatory IL-6 cytokine and regulatory IL-10 cytokine (Figure 4).

Spearman correlation analysis of data on several demographic parameters, IgG subclass, and spontaneous cytokine production in PBMCs from patients in the first cohort is presented in the Supplementary Data (Figure S3) and shows a moderately positive relationship between age and the spontaneous production of IL-1β, TNF-α, IFN-γ, and IL-12P70 (Figure S3). A significant negative association was found between sex and TGF-β release, with women showing statistically significantly higher levels of spontaneous TDF-β production than men (Figure S4). This cytokine release was also associated with a moderately positive correlation with previous COVID-19 infection, while there was a moderate to significant negative correlation with IgG2 levels to N and S-proteins. Previous vaccination with coronavirus vaccines did not correlate with any of these parameters. IgG3 to the N-protein correlated moderately positively with IL-2 and TNF-α, and anti-S IgG3 also correlated moderately positively with TNF-α (Figure S3).

**Figure 5:**
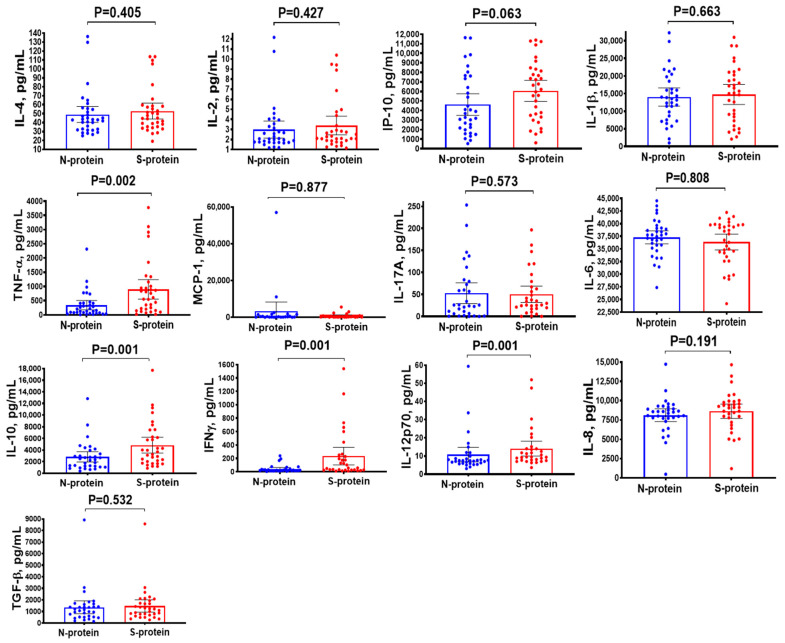
Production of cytokines by PBMCs from patients of the 1st cohort (*n* = 33), upon stimulation with recombinant N- and S-protein. The statistical significance was determined using Wilcoxon matched-pairs signed rank test.

Stimulation of PBMCs with S-protein from SARS-CoV-2 led to a significant increase in production of most cytokines compared to N-protein (Figure 5), while the most pronounced increase in production levels is observed for IL-10, TNF-α, IFN-γ, and IL-12p70 (Figure 5).

**Figure 6:**
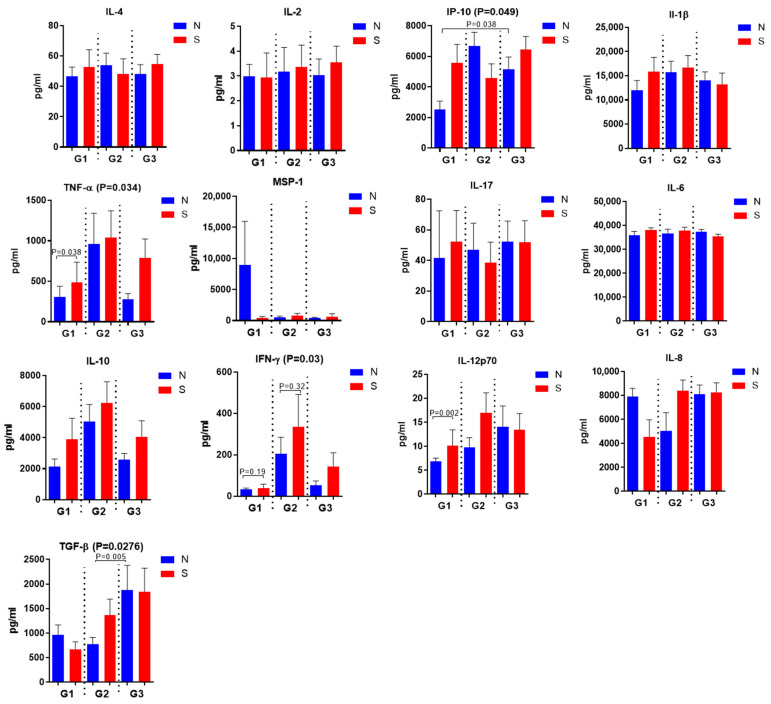
Production of cytokines by PBMCs upon stimulation with recombinant N- and S-protein from different patient groups. G1—not vaccinated, *n* = 8, G2—no COVID-19, vaccinated, *n* = 9; G3—prior COVID-19, vaccinated (*n* = 16). *p* values were calculated using the Kruskal-Wallis test for multiple comparisons and the Mann-Whitney test for pairwise comparisons.

Among those who did not have COVID-19 but have been vaccinated, levels of IL-4, IP-10, IL-17 to the S-protein are reduced (Figure 6). Among those who had COVID-19 and have been vaccinated, there are lower levels of IL-1-β, IL-6 and IL-12p70 in response to S-protein compared to stimulation of N-protein (Figure 6).

## 4. Discussion

The present study provides a comprehensive analysis of long-term humoral and cellular immune responses to SARS-CoV-2 in individuals with different exposure histories, including prior infection, vaccination, or combined exposure. In contrast to most studies focusing on early immune responses, our analysis extends the observation period to several years after antigen exposure, providing insight into long-term immune persistence. The results demonstrate that virus-specific IgG antibodies remain detectable for prolonged periods and that antibodies directed against the RBD of the spike protein correlate with virus-neutralizing activity measured in an in vitro model. Stimulation of PBMCs with recombinant viral antigens demonstrated sustained functional responsiveness of T-cell populations, indicating preserved immunological memory. The enhanced immune responses observed in individuals with hybrid immunity are consistent with previous reports showing increased magnitude and breadth of adaptive immune responses following combined exposure [26,27]. Long-term antibody persistence after SARS-CoV-2 infection is thought to be due to the formation of long-lived plasma cells in the bone marrow, supporting the concept of memory B cells in the bone marrow that produce long-lived antibodies [28].

The observed predominance of IgA antibodies in vaccinated individuals supports the notion that vaccination induces an immunoglobulin profile distinct from that of natural infection. The difference likely reflects the differences in antigen presentation and the immune priming provided by vaccine platforms that express the S-protein. Importantly, the lack of virus-specific IgM in all examined groups suggests that the observed immune responses are indicative of immunological memory, rather than active infection [29]. At the cellular level, we observed sustained functional activity of peripheral blood mononuclear cells in individuals with prior SARS-CoV-2 exposure. PBMCs from patients who recovered from COVID-19 and subsequently received vaccination demonstrated the highest frequency of memory T-helper cells and cytotoxic T-cell populations that were responsive to antigens. This finding supports the concept of hybrid immunity, in which sequential exposure to viral antigens through infection and vaccination results in enhanced magnitude and breadth of adaptive immune responses [30]. The biological mechanisms underlying hybrid immunity include a 5- to 10-fold increase in high-quality memory B cells, enhanced affinity maturation of antibodies that broadens their ability to neutralize variants, and the complementary imprinting of the immune system to target multiple viral antigens simultaneously. Additionally, more robust and polyfunctional T cell responses provide superior protection against severe outcomes [4,5].

A key finding of this study was the differential immune activation induced by the recombinant S and N-proteins in vitro. The stronger cytokine response induced by the S-protein reflects its central role in antiviral immunity and supports its continued use as a primary target in vaccine design. The S-protein stimulated a more pronounced cytokine response than the N-protein, including an increase in the production of IFN-gamma, TNF-alpha, and IL12p70 cytokines. These cytokines characterize Th1-like immune responses and the activation of cytotoxic T cells, which play an important role in antiviral defense [9,28]. At the same time, the S-protein stimulation was accompanied by an increase in IL-10 production, indicating the activation of regulatory pathways that may help limit excessive inflammation and preserve immune balance [31,32].

Notably, individuals who have experienced both COVID-19 and received vaccination exhibited prolonged spontaneous cytokine production from PBMCs, including IL-2 and IL-17A, suggesting sustained functional readiness of the T-cell populations [33]. The observed increase in regulatory cytokines such as IL-10 and TGF-β suggests the activation of compensatory mechanisms that may prevent excessive inflammation and maintain immune homeostasis [34]. Similar mixed effector–regulatory cytokine profiles have been described in studies analyzing cytokine-producing capacity in PBMCs from COVID-19 patients following nonspecific or antigen-specific stimulation [34,35,36]. Elevated spontaneous intracellular TGF-β production observed in post-COVID individuals reflects long-term immune remodeling after natural infection [36,37]. In individuals with persistent anti-N IgM and limited cross-reactivity to XBB.1.5 RBD, increased TGF-β levels may indicate regulatory constraints on B cell maturation, potentially contributing to prolonged IgM persistence and sub-optimal variant-adapted humoral immunity [38]. In our study, none of the participants had serum IgM, and, in this case, elevated spontaneous intracellular TGF-β production likely reflects long-term regulatory immune remodeling following SARS-CoV-2 infection rather than ongoing immune activation. Importantly, no participants in the study exhibited clinical symptoms of post-COVID or post-vaccine inflammatory conditions at time of sampling. Therefore, prolonged immune activation observed is more likely due to adaptive immune remodeling rather than pathological chronic inflammation. Higher TGF-β levels in women may be due to sex-specific differences in immune response, but further investigation is needed in female patients.

In the context of differentiating between individuals with or without prior SARS-CoV-2 infection, antibodies directed against the N-protein remain useful as serological markers. Specifically, elevated IgG3 levels against the N-protein may indicate natural infection, in line with previous findings that anti-N antibody levels do not significantly affect virus neutralization, but do reliably reflect previous viral exposure [26,27]. The nanoscale formulation of the vaccine can affect proinflammatory cytokine levels [39].

This study has several limitations that should be considered when interpreting the results. One limitation of the study is that at the time of the investigation, there were no immunologically naïve individuals. Determining the prior SARS-CoV-2 infection status is challenging because many infections remain asymptomatic or go undocumented. Our study was based on medical history and available clinical documentation, but we acknowledge that unreported infections cannot be excluded completely. Due to the cross-sectional design, the study does not allow assessment of intra-individual immune persistence or temporal trajectories. The term “long-term” in this context means the detection of immune responses in individuals examined several years after exposure rather than continued immune maintenance in the same individuals over time. While longitudinal studies would be needed to define individual immune trajectories, cross-sectional analysis at extended post-exposure time points provides valuable information about the range and variation of immune parameters present in the population several years after antigen exposure.

## 5. Conclusions

Overall, our findings suggest that SARS-CoV-2 infections and vaccinations induce long-term and functionally effective immune responses, characterized by detectable antibody responses at later time points and persistent cellular responsiveness. IgG antibodies persist for several years post-infection or vaccination. Antibodies to the RBD of the virus persist for several years after infection or vaccination, and correlate strongly with neutralizing activity against the virus. Antibodies against the N-protein indicate prior infection, but do not necessarily neutralize the virus. Vaccinated individuals show a higher prevalence of IgA antibodies. No IgM antibodies were found among the patients examined.

PBMCs from individuals who have been infected and vaccinated show enhanced responsiveness, and there are more memory T-cells. Hybrid immunity, resulting from combined infection and vaccination, provides broader and more robust immune responses. With high levels of population-wide hybrid immunity, the focus of vaccine evaluation has shifted from preventing any infection to preventing severe outcomes, hospitalization, and long COVID-19.

Stimulation with S-protein induces stronger production of cytokines (IFN-γ, TNF-α, IL-12p70) than N-protein. Regulatory cytokines such as IL-10 and TGF-β are also elevated, suggesting immune regulation, rather than chronic inflammation. The S-protein plays a central immunostimulatory role, highlighting its importance as a key target for vaccine development. Long-lasting immunity provides durable immune memory, but there are differences between infection responses and vaccination that highlight the importance of tailored immunization approaches.

Future research should focus on long-term effects, hybrid immunity, and optimizing other vaccine types, in addition to Adenovector vaccines, such as multi-antigen and chimeric protein-based vaccines.

## Figures and Tables

**Table 1:** Study design and definition of study groups.

Cohort	Group	Infection Status	Vaccination Status	Time Since Infection	Males/Females	Inclusion Criteria
Cohort 1 (*n* = 43)	Group 1 (*n* = 10)	Mostly prior to COVID-19	Not vaccinated	Median 36 (Q25–Q75: 13.5–50.3 months)	3 (30%)/7 (70%)	Adults vaccinated with Gam-COVID-Vac (Sputnik V), with or without prior COVID-19
	Group 2 (*n* = 14)	No documented COVID-19	Vaccinated	Non applicable	8 (57.1%)/6 (42.9%)	Adults vaccinated against SARS-CoV-2 with no documented history of COVID-19
	Group 3 (*n* = 19)	Prior to COVID-19	Vaccinated	Median 48.5 months (Q25–Q75: 36–56.5 months) since infection	6 (31.6%)/13 (38.4%)	Adults with documented history of COVID-19 and subsequent vaccination
Cohort 2 (*n* = 32)	Unvacci-nated (*n* = 12)	Prior to COVID-19	Not vaccinated	Median 22 months (Q25–Q75: 13.25–24.75 months) since infection	1 (8.3%)/13 11(91.7%)	Adults previously infected with SARS-CoV-2 who had not received vaccination
	Vaccinated (*n* = 20)	Mostly prior to COVID-19	Vaccinated	Median 18 months (Q25–Q75: 13–31.5 months) since infection	4 (20%)/16 (80%)	Adults vaccinated with Gam-COVID-Vac (Sputnik V), with or without prior COVID-19

**Table 2:** Molecular structure and epitope analysis of recombinant proteins. Predicted epitopes are highlighted in red, while non-epitope regions are marked in gray.

Name	Analysis of Epitopes Compatible with Human MHC-I	Spatial Structure
SARSN1	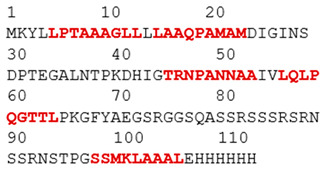	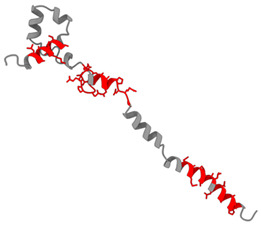 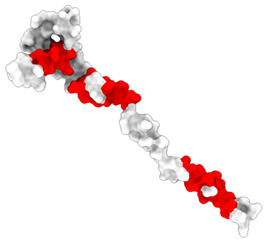
S-SARS “XBB.1.5”	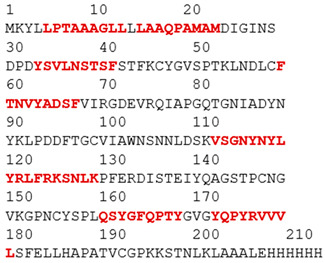	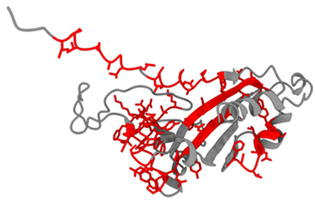 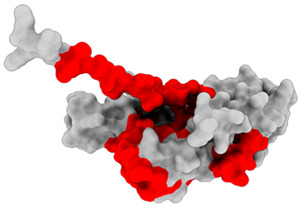

**Table 3:** Recombinant protein SARSN1.

No.	Epitope (Location in the Amino Acid Sequence of a Protein)	Allele
1	LPTAAAGLL (5–13)	HLA-B0702
2	LAAQPAMAM (15–23)	HLA-B0702
3	TRNPANNAA (45–53)	HLA-B3901
4	LQLPQGTTL (56–64)	HLA-B3901
5	SSMKLAAAL (98–106)	HLA-B3901

**Table 4:** Recombinant protein S-SARS”XBB.1.5” (Kraken).

No.	Epitope (Location in the Amino Acid Sequence of a Protein)	Allele
1	LPTAAAGLL (5–13)	HLA-B0702
2	LAAQPAMAM (15–23)	HLA-B0702
3	YSVLNSTSF (33–41)	HLA-B5801, HLA-B1501
4	FTNVYADSF (59–67)	HLA-B5801
5	VSGNYNYLY (112–120)	HLA-A0101
6	NYNYLYRLF (115–123)	HLA-A2402
7	YRLFRKSNL (120–128)	HLA-B2705
8	RLFRKSNLK (121–129)	HLA-A0301
9	QSYGFQPTY (160–170)	HLA-B5801
10	YQPYRVVVL (172–180)	HLA-B3901
11	PYRVVVLSF (174–182)	HLA-A2402
12	TNLKLAAAL (198–206)	HLA-B3901

**Table 5:** Memory T-helper cells (1st cohort, *n* = 43).

Cell Populations	Cell Subpopulations	Group					
		Not Vaccinated (Group 1)		No COVID-19, Vaccinated (Group 2)		COVID-19, Vaccinated (Group 3)	
		# In Group					
		10		14		19	
		SARS-CoV-2 Proteins					
		N	S	N	S	N	S
General pool of T-helper memory cells (CD3 + CD4 + CD45RA-)	IFNg+	4 (40%)	7 (70%)	8 (57%)	5 (36%)	13 (68%)	13 (68%)
	IL-2+	5 (50%)	7 (70%)	7 (50%)	9 (64%)	12 (63%)	15 (79%)
	TNFa+	6 (60%)	6 (60%) ^1^	10 (71%)	12 (86%)	15 (79%)	18 (95%)
	IFN + TNF+	5 (50%)	4 (40%)	5 (36%)	3 (21%)	9 (47%)	13 (68%)
	polyfunc	2 (20%)	2 (20%) ^2^	3 (21%)	4 (29%) ^3^	7 (37%) ^4^	14 (74%)
T-helper cells of central memory (CD45RA-CCR7+)	IFNg+	1 (10%) ^5^	7 (70%)	2 (14%)	5 (36%)	6 (32%)	7 (37%)
	IL-2+	5 (50%)	6 (60%)	5 (36%)	8 (57%)	11 (58%)	10 (53%)
	TNFa+	6 (60%)	6 (60%)	9 (64%)	10 (71%)	15 (79%)	16 (84%)
	IFN + TNF+	3 (30%)	1 (10%)	1 (7%)	1 (7%)	3 (16%)	4 (21%)
	polyfunc	0 (0%)	0 (0%)	0 (0%)	0 (0%)	3 (16%)	3 (16%)
Effector memory T-helper cells (CD45RA-CCR7-)	IFNg+	6 (60%)	5 (50%)	9 (64%)	7 (50%)	12 (63%)	13 (68%)
	IL-2+	4 (40%)	5 (50%)	7 (50%)	7 (50%)	11 (58%)	16 (84%)
	TNFa+	6 (60%)	6 (60%)	10 (7%)	11 (79%)	15 (79%)	17 (90%)
	IFN + TNF+	5 (50%)	4 (40%)	4 (29%)	3 (21%)	8 (42%)	11 (58%)
	polyfunc	2 (20%)	2 (20%) ^6^	3 (21%)	4 (29%)	8 (42%)	13 (68%)

^1^—The number of S-protein responders in Group 1 was lower than in Group 3 (*p* < 0.05, Fisher’s exact test); ^2^—The number of S-protein responders in Group 1 was lower than in Group 3 (*p* < 0.05, Fisher’s exact test); ^3^—The number of S-protein responders in Group 2 was lower than in Group 3 (*p* < 0.05, Fisher’s exact test); ^4^—The number of N-protein responders was lower than S-protein responders (*p* < 0.05, Fisher’s exact test); ^5^—The number of N-protein responders was lower than S-protein responders (*p* < 0.01, Fisher’s exact test); ^6^—The number of N-protein responders was lower than S-protein responders (*p* < 0.05, Fisher’s exact test).

**Table 6:** Cytotoxic CD8+ memory T cells (1st cohort, *n* = 43).

Cell Populations	Cell Subpopulations	Group					
		Not Vaccinated (Group 1)		No COVID-19, Vaccinated (Group 2)		COVID-19, Vaccinated (Group 3)	
		# In Group					
		10		14		19	
		SARS-CoV-2 Proteins					
		N	S	N	S	N	S
Total pool of cytotoxic CD8+ memory T cells (CD3 + CD8 + CD45RA-)	IFNg+	2 (20%)	4 (40%)	5 (36%)	4 (29%)	7 (37%)	3 (16%)
	IL-2+	3 (30%)	3 (30%)	4 (29%)	4 (29%)	9 (47%)	9 (47%)
	TNFa+	6 (60%)	5 (50%) ^1^	13 (93%)	11 (79%)	16 (84%)	17 (90%)
	IFN + TNF+	0 (0%)	1 (10%)	1 (7%)	0 (0%)	1 (5%)	1 (5%)
	polyfunc	0 (0%)	0 (0%)	0 (0%)	0 (0%)	0 (0%)	1 (5%)
Cytotoxic CD8+ T cells of central memory (CD45RA-CCR7+),	IFNg+	1 (10%)	1 (10%)	1 (7%)	1 (7%)	3 (16%)	1 (5%)
	IL-2+	4 (40%)	3 (30%)	2 (14%)	4 (29%)	4 (21%)	6 (32%)
	TNFa+	6 (60%)	5 (50%)	6 (43%)	4 (29%)	11 (58%)	10 (53%)
	IFN + TNF+	0 (0%)	0 (0%)	0 (0%)	0 (0%)	0 (0%)	0 (0%)
	polyfunc	0 (0%)	0 (0%)	0 (0%)	0 (0%)	0 (0%)	0 (0%)
Cytotoxic CD8+ T cells of effector memory (CD45RA-CCR7-)	IFNg+	2 (20%)	4 (40%)	5 (36%)	5 (36%)	5 (26%)	2 (11%)
	IL-2+	2 (20%)	2 (20%)	4 (29%)	4 (29%)	8 (42%)	9 (47%)
	TNFa+	5 (50%)	5 (50%)	11 (79%)	9 (64%)	13 (68%)	14 (74%)
	IFN + TNF+	3 (30%)	3 (30%)	7 (50%)	7 (50%)	9 (47%)	9 (47%)
	polyfunc	0 (0%)	0 (0%)	0 (0%)	0 (0%)	0 (0%)	0 (0%)

^1^—The number of patients who responded to S-protein stimulation in Group 1 was lower than in Group 3 (*p* < 0.05, Fisher’s exact test).

## Data Availability

All data are presented in the text of the article.

## Supplementary Materials

The following supporting information can be downloaded at: https://www.mdpi.com/article/10.3390/biomedicines14040923/s1, Figure S1. Flow cytometry immunophenotyping gating strategy for antigen-specific CD4+ and CD8+ T cell subsets. Figure S2. Cytokine production by antigen-specific CD4+ and CD8+ T cell subsets. Figure S3. Spearman correlations of several demographic parameters, various IgG subclasses and cytokine production by peripheral blood mononuclear cells among examined patients from Cohort 1 (*n* = 33). The presence of a statistical relationship between the variables was estimated via a correlation analysis in Python 3 using the Pandas library ‘Corr’ function using Spearman’s method. The *p*-value < 0.05 was considered to be statistically significant. Class variables were transformed into numeric ones as follows: “Sex: Female—1, Male—2; COVID-19 history—1, no COVID-19—0; coronavirus vaccination: yes—1, no—0. The strength of the relationship was assessed using the Chaddock scale: from 0 to 0.3—weak; from 0.3 to 0.5—moderate; from 0.5 to 0.7—noticeable; from 0.7 to 0.9—high; from 0.9 to 1—very high. Figure S4. Spontaneous production of free active TGF-β Spontaneous production of free active mononuclear cells in peripheral blood in men and women.

## Abbreviations

The following abbreviations are used in this manuscript:

CI	confidence interval
CPE	cytopathic effect
COVID-19	coronavirus disease 19
IFN-γ	interferon-γ
IL	interleukin
MCP-1	monocyte chemotactic protein
Me	medians
N-protein	Nucleocapsid coronavirus protein
Q1; Q3	lower and upper quartiles
PBMCs	peripheral mononuclear cells
SARS-CoV-2	severe acute respiratory syndrome coronavirus 2
S-protein	Spike coronavirus protein
TGF-β	transforming growth factor-beta
TNF-α	tumor necrosis factor alpha
