# Supplementary material for: The Effects of Past COVID-19 and Vaccination on Antibody Levels, Cellular Immunity, and Cytokine Production by Peripheral Blood Mononuclear Cells
Source: Biomedicines. 2026 Apr 17;14(4):923. doi: 10.3390/biomedicines14040923 (PMC13113839; doi:10.3390/biomedicines14040923)
Supplement: Supplementary file 1 [file biomedicines-14-00923-s001.zip › biomedicines-4192743-supplementary.pdf]

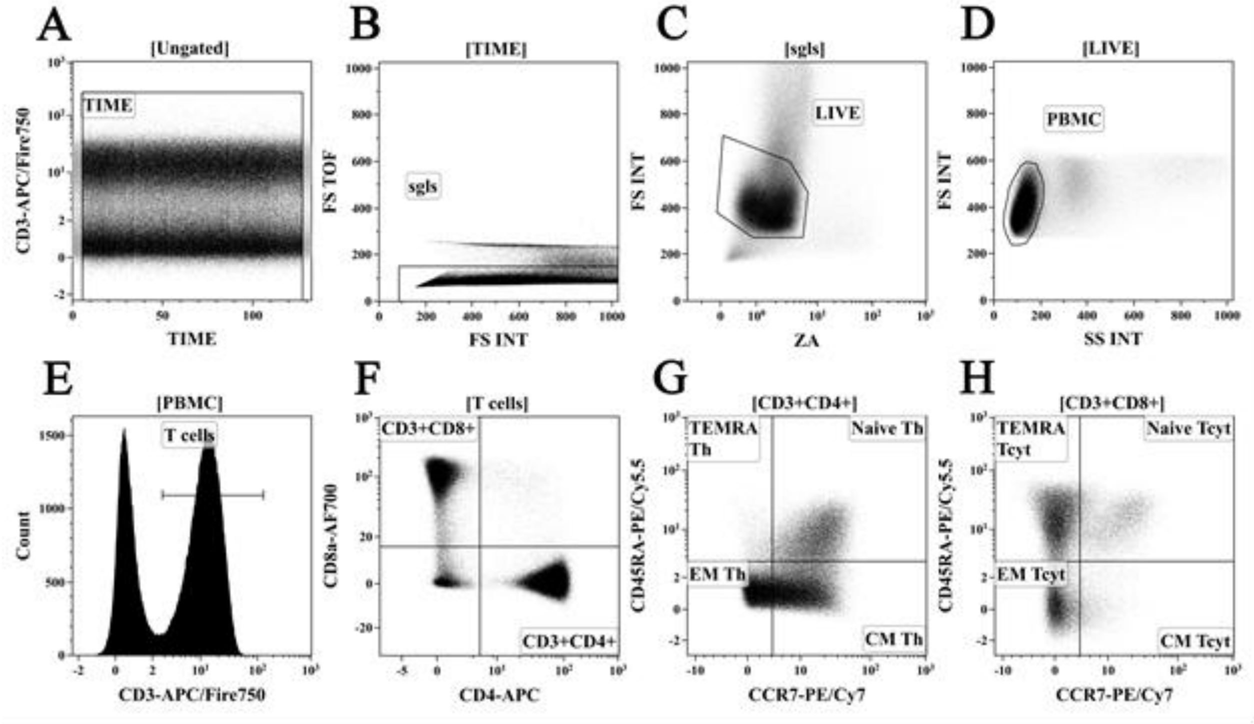

Figure S1. Flow cytometry immunophenotyping gating strategy for antigen-specific CD4+ and CD8+ T cell subsets.

Dot plot (A) – artifact exclusion time gating; dot plot (B) – doublets exclusion from the analysis using the ratio between integral and peak forward scatter signals; dot plot (C) – dead cells exclusion from the analysis using Zombie Aqua staining; dot plot (D) – total lymphocyte subset purification based on side scatter and forward scatter; dot plot (E) – total T cell subset gaiting based on CD3 expression; dot plot (F) – detection of CD4+ and CD8+ T cells within total CD3+ T cell subset; dot plot (G) and (F) – four main CD4+ and CD8+ T cell maturation subsets were identified, including 'naïve" cells (CD45RA+CCR7+), central and effector memory cells (CD45RA–CCR7+ and CD45RA–CCR7–, respectively), as well as TEMRA cells with CD45RA+CCR7– phenotype.

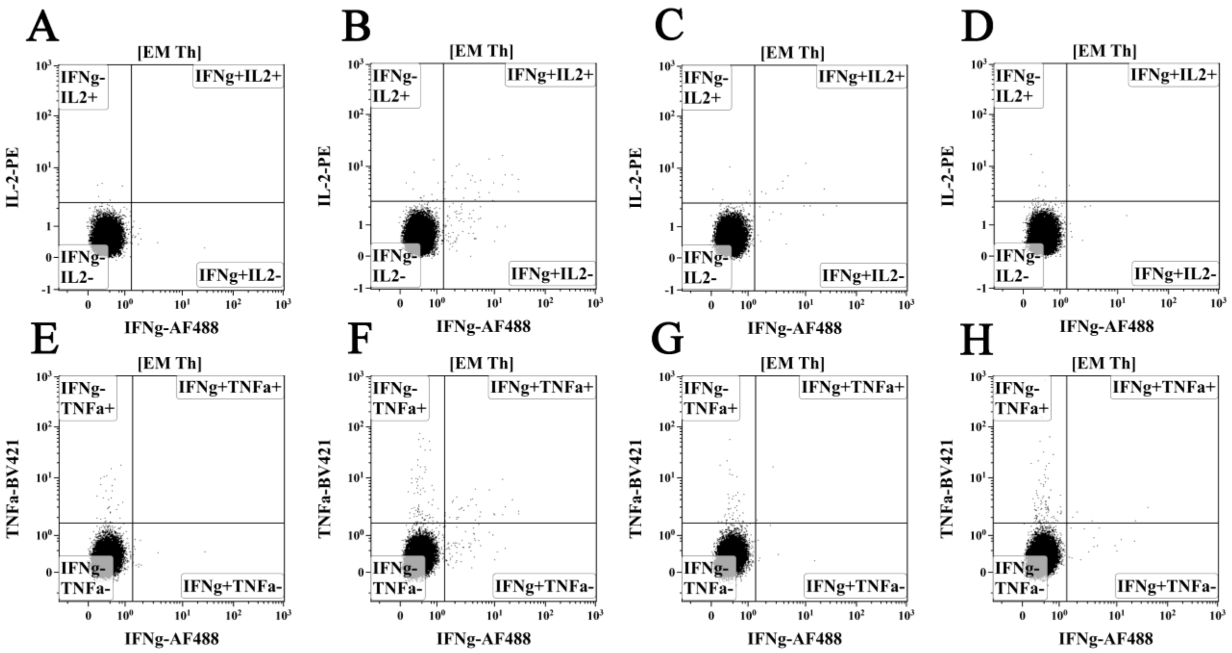

Figure S2. Cytokine production by antigen-specific CD4+ and CD8+ T cell subsets.

Representative dot plots showing intracellular cytokine production by effector memory CD45RA–CCR7– Th cells in negative control samples (dot plots A and E), PPD-stimulated controls (positive or biological controls, dot plots B and F), cell samples stimulated with N-protein and S-protein (dot plots C and G and dot plots D and H, respectively).

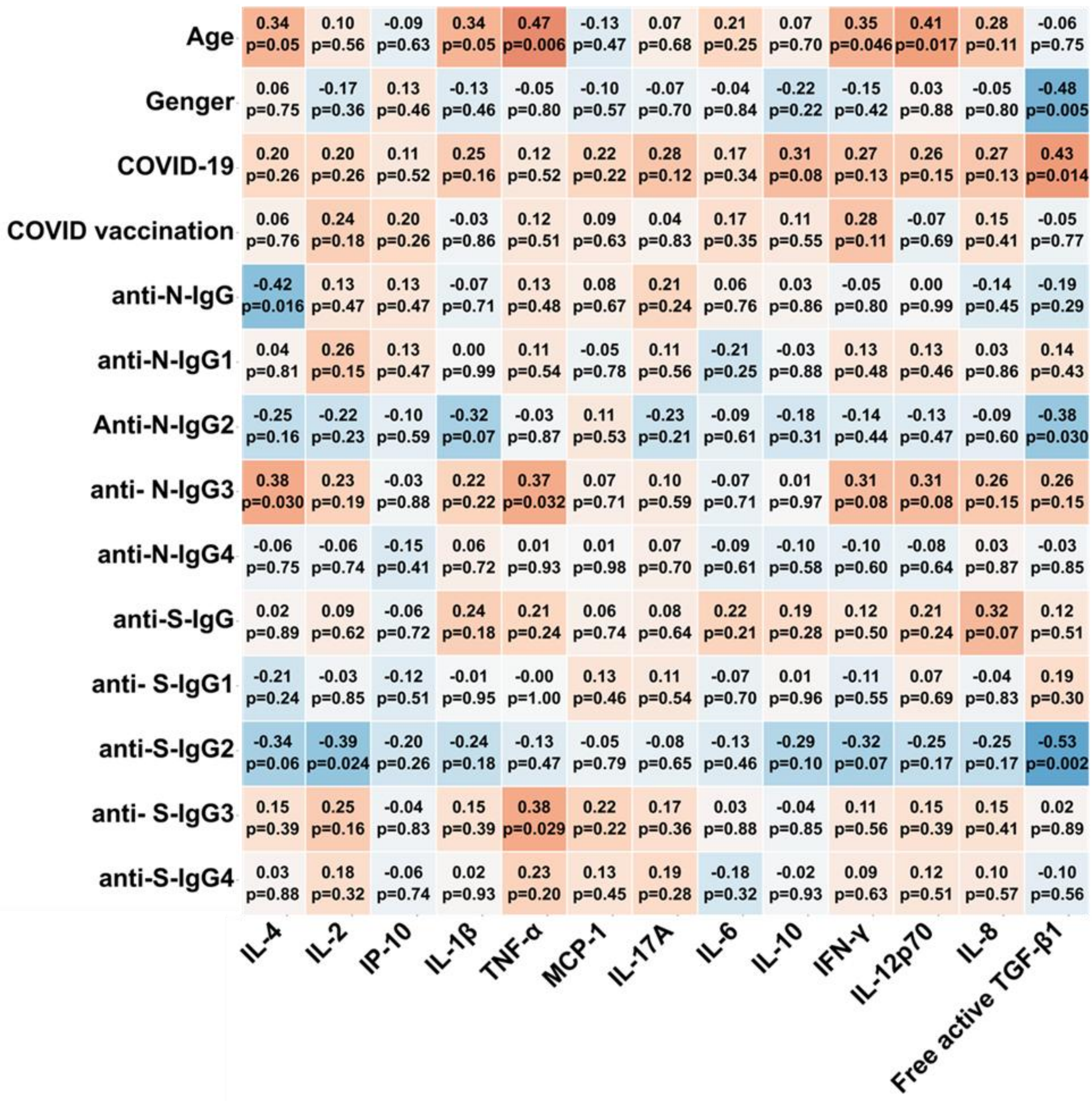

Figure S3. Spearman correlations of several demographic parameters, various IgG subclasses and cytokine production by peripheral blood mononuclear cells among examined patients from Cohort 1 (n=33). The presence of a statistical relationship between the variables was estimated via a correlation analysis in Python 3 using the Pandas library ‘Corr’ function using Spearman’s method. The p-value < 0.05 was considered to be statistically significant. Class variables were transformed into numeric ones as follows: “Sex: Female - 1, Male - 2; COVID-19 history - 1, no COVID-19 - 0; coronavirus vaccination: yes - 1, no – 0. The strength of the relationship was assessed using the Chaddock scale: from 0 to 0.3 - weak; from 0.3 to 0.5 - moderate; from 0.5 to 0.7 - noticeable; from 0.7 to 0.9 - high; from 0.9 to 1 - very high.

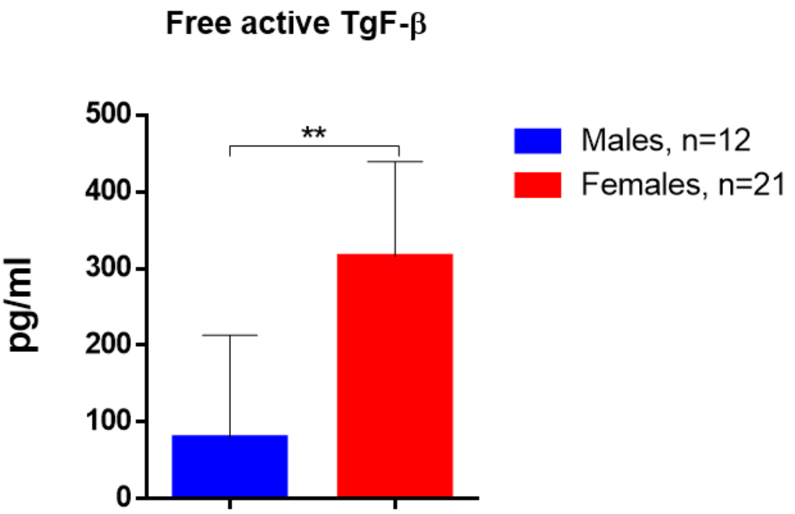

Figure S4. Spontaneous production of free active TGF-β Spontaneous production of free active mononuclear cells in peripheral blood in men and women.
